# Mechanisms of Immunopathogenesis in Cutaneous Leishmaniasis And Post Kala-azar Dermal Leishmaniasis (PKDL)

**DOI:** 10.3389/fcimb.2021.685296

**Published:** 2021-06-08

**Authors:** Greta Volpedo, Thalia Pacheco-Fernandez, Erin A. Holcomb, Natalie Cipriano, Blake Cox, Abhay R. Satoskar

**Affiliations:** ^1^ Department of Pathology, The Ohio State University Wexner Medical Center, Columbus, OH, United States; ^2^ Department of Microbiology, College of Arts and Sciences, The Ohio State University, Columbus, OH, United States

**Keywords:** Th1/Th2, Cutaneous leishmaniasis, post Kala-azar dermal leishmaniasis, immunoregulation, immunopathology

## Abstract

Leishmaniasis is a neglected tropical disease that affects 12 million people worldwide. The disease has high morbidity and mortality rates and is prevalent in over 80 countries, leaving more than 300 million people at risk of infection. Of all of the manifestations of this disease, cutaneous leishmaniasis (CL) is the most common form and it presents as ulcerating skin lesions that can self-heal or become chronic, leading to disfiguring scars. This review focuses on the different pathologies and disease manifestations of CL, as well as their varying degrees of severity. In particular, this review will discuss self-healing localized cutaneous leishmaniasis (LCL), leishmaniasis recidivans (LR), mucocutaneous leishmaniasis (MCL), anergic diffuse cutaneous leishmaniasis (ADCL), disseminated leishmaniasis (DL), and Post Kala-azar Dermal Leishmaniasis (PKDL), which is a cutaneous manifestation observed in some visceral leishmaniasis (VL) patients after successful treatment. The different clinical manifestations of CL are determined by a variety of factors including the species of the parasites and the host’s immune response. Specifically, the balance between the pro and anti-inflammatory mediators plays a vital role in the clinical presentation and outcome of the disease. Depending upon the immune response, *Leishmania* infection can also transition from one form of the disease to another. In this review, different forms of cutaneous *Leishmania* infections and their immunology are described.

## Introduction

Leishmaniasis is one of the 17 Neglected Tropical Diseases (NTDs) as defined by the World Health Organization (WHO) (Centers for Disease Control and Prevention, 2020; World Health Organization 2021a). NTDs are endemic in tropical and sub-tropical countries with limited access to healthcare, and where a large portion of the population lives in close contact with disease vectors and reservoirs (Harhay et al., 2011; Millán et al., 2014). Leishmaniasis affects 12 million people worldwide, with over 300 million individuals at risk of contracting the infection (Pavli and Maltezou, 2010). Despite the significant morbidity and mortality of leishmaniasis around the globe, there is currently no prophylactic vaccine and the available therapeutics present significant challenges, including toxicities, poor patience compliance, and the development of parasitic resistance (McGwire and Satoskar, 2014).

Leishmaniasis is caused by over 21 species of the genus *Leishmania*, a protozoan parasite transmitted by the sand fly vector (Pace, 2014). During a blood meal, flagellated promastigotes deposited into the dermis are engulfed by phagocytes such as neutrophils and macrophages, where they transform into amastigotes, a stage of the parasite better equipped to deal with the temperature and pH changes. Amastigotes replicate inside the phagocyte until they rupture the cell and infect other tissues (McGwire and Satoskar, 2014).

*Leishmania* parasites cause a wide range of diseases, such as cutaneous (CL), diffuse cutaneous (DCL), mucosal (MCL) and visceral (VL) leishmaniasis (Pace, 2014). Cutaneous leishmaniasis (CL) is the most common form, which is characterized by skin lesions that can ulcerate and leave disfiguring scars, a source of discrimination in the affected communities (Okwor and Uzonna, 2016a; World Health Organization 2021b). Although CL is not lethal, it can lead to disabilities that can affect an individual’s ability to work and lead a normal life. The impact of this disease has significantly increased in the past few decades; in particular, the disability adjusted life years (DALYs) from CL rose 43.5% between 1990 and 2016 (Hotez, 2018).

Cutaneous *Leishmania* infections can manifest as self-healing localized cutaneous leishmaniasis (LCL), leishmaniasis recidivans (LR), mucocutaneous leishmaniasis (MCL), anergic diffuse cutaneous leishmaniasis (ADCL), and disseminated leishmaniasis (DL) (Scorza et al., 2017). Furthermore, Post Kala-azar Dermal Leishmaniasis (PKDL) is a dermatological complication of VL, which occurs in some VL patients in Africa and Asia within a year after completion of WHO recommended treatment (McGwire and Satoskar, 2014). Transmission of *Leishmania* spp. specific to each disease form is carried out by 98 species of phlebotomine sandflies coming from the genus *Phlebotomus* and *Lutzomyia*. In the Old World, 42 *Phlebotomus* species allow for the transmission of *L. infantum, L. donovani, L. major, L. tropica*, and *L. aethiopica*. In almost all cases, each species of *Phlebotomus* transmits only one species of *Leishmania*. In the New World, 56 *Lutzomyia* species transmit 15 species of *Leishmania*, including *L. infantum (= Leishmania chagasi), Leishmania guyanensis, Leishmania mexicana, Leishmania amazonensis, Leishmania braziliensis*, and *Leishmania panamensis.* Contrary to the genus *Phlebotomus*, *Lutzomyia* spp. have been shown to transmit more than one species of *Leishmania*. Maroli et al. provides an extensive review of the numerous sandfly species associated with *Leishmania* spp., which we encourage the reader to reference (Maroli et al., 2013).

**Figure 1:**
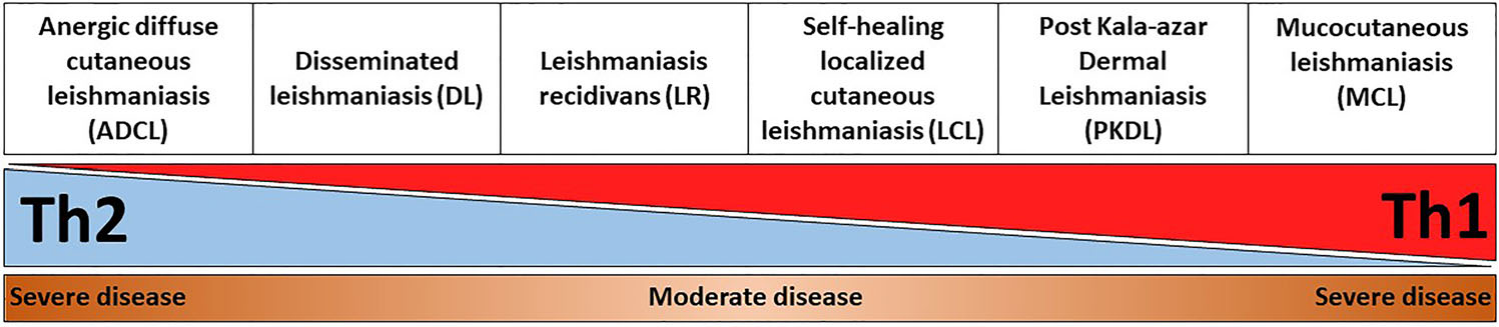
Representation of disease severity and Th1-Th2 balance across different CL and PKDL forms. An exaggerated Th1, or alternatively Th2, response is equally detrimental for leishmaniasis patients as it results in enhanced disease severity. For instance, an uncontrolled Th2 response in anergic diffuse cutaneous leishmaniasis (ADCL) can lead to cell anergy and increased disease pathology. On the other hand, an unrestrained Th1 response in mucocutaneous leishmaniasis (MCL) can cause sustained inflammation and tissue damage.

This review describes in detail different clinical forms of cutaneous *Leishmania* infections and PKDL, with a special focus on the unique immunological signatures that are associated with these diseases. In particular, a balance between a Th1 and Th2 response is necessary for controlling the infection. An exaggerated polarization towards Th1 or Th2 leads to severe disease pathology (**Figure 1**). Understanding the immunological mechanisms that are responsible for resolution or pathogenesis of different clinical forms of CL and PKDL will allow development of the next generation of therapeutics and vaccines against leishmaniasis.

## Self-Healing Localized Cutaneous Leishmaniasis (LCL)

### Epidemiology

The most common clinical manifestation of leishmaniasis is Localized Cutaneous Leishmaniasis (LCL). More than 70% of LCL cases worldwide are reported in ten countries: Afghanistan, Algeria, Brazil, Colombia, Costa Rica, Ethiopia, Iran, Syria, North Sudan, and Peru (Alvar et al., 2012). LCL is commonly caused by *L. major*, *L. tropica*, and *L. aethiopica* in the Old World, but it can also be due to *L. infantum* (Steverding, 2017). Interestingly, in Sri Lanka LCL can also be caused by a local *L. donovani* strain, which is genetically different from other *L. donovani* strains (Kariyawasam et al., 2017; Siriwardana et al., 2018). In the New World, LCL is caused by *L. amazonensis, L. mexicana*, *L. venezuelensis*, and *L. braziliensis* (Steverding, 2017) (**Table 1**).

**Table 1:** Geographical distribution and causative species of different CL and PKDL forms.

CL/PKDL form	*Leishmania* causative species	Geographical location
**Self-healing localized cutaneous leishmaniasis (LCL)**	**OLD WORLD:**	**OLD WORLD:**
	*L. major*	Afghanistan, Algeria, Ethiopia, Iran, North Sudan, Syria, Sri Lanka
	*L. tropica*	**NEW WORLD:**
	*L. aethiopica*	Brazil, Colombia, Costa Rica, Peru
	*L. donovani*	
	*L. infantum*	
	**NEW WORLD:**	
	*L. amazonensis*	
	*L. mexicana*	
	*L. venezuelensis*	
	*L. braziliensis*	
**Leishmaniasis recidivans (LR)**	**OLD WORLD:**	**OLD WORLD:**
	*L. major*	Ethiopia, India, Pakistan
	*L. tropica*	**NEW WORLD:**
	**NEW WORLD:**	Brazil
	*L. braziliensis*	
	*L. amazonensis*	
	*L. panamensis*	
	*L. guyanensis*	
**Mucocutaneous leishmaniasis (MCL)**	**OLD WORLD:**	**OLD WORLD:**
	*L. major*	Ethiopia, Iran, Sudan
	*L. tropica*	**NEW WORLD:**
	*L. aethiopica*	Bolivia, Brazil, Peru
	*L. donovani*	
	*L. infantum*	
	**NEW WORLD:**	
	*L. braziliensis*	
	*L. amazonensis*	
	*L. panamensis*	
	*L. guyanensis*	
**Anergic diffuse cutaneous leishmaniasis (ADCL)**	**OLD WORLD:**	**OLD WORLD:**
	*L. aethiopica*	Ethiopia and Kenya, Namibia, Tanzania
	**NEW WORLD:**	**NEW WORLD:**
	*L. mexicana*	Brazil, Bolivia, Colombia, Dominican Republic, Ecuador, Honduras, Mexico, Nicaragua, Peru, U.S.A. (Texas), and Venezuela
	*L. amazonensis*	
**Disseminated leishmaniasis (DL)**	**NEW WORLD:**	**NEW WORLD:**
	*L. braziliensis*	North-east Brazil, Colombia
	*L. panamensis*	
	*L. guyanensis*	
	*L. amazoniensis*	
**Post Kala-azar Dermal Leishmaniasis (PKDL)**	**OLD WORLD:**	**OLD WORLD:**
	*L. donovani*	East Africa: Ethiopia, Kenya, Sudan, Uganda
		South East Asia: Bangladesh, India, Nepal

LCL is characterized by the formation of slow healing skin lesions at or near the site of the bite of the infected sand fly. Lesions begin as small red papules and develop into painless nodules, which normally ulcerate to form circumscribed ulcers (Dowlati, 1996). These lesions can get secondarily infected with bacteria or fungi, most commonly with Staphylococcal species (Ziaei et al., 2008), and can be mistaken for skin lesions caused by other infectious and non-infectious skin ailments (Alfahaad, 2020). The onset of clinical symptoms and lesion development in the host occurs after an asymptomatic incubation period ranging from a few days to 3 years, but typically lasting 2-8 weeks (Scorza et al., 2017). The parasites remain localized to the infection site during this time, so that each lesion represents an independent sand fly bite. These infection sites are usually found on exposed sites of the body such as the arms, legs, and face (Maurer et al., 2009).

LCL lesions self-heal without treatment (Scorza et al., 2017) in 0% to over 70% of patients (Morizot et al., 2013), depending on the immune response and the species of *Leishmania* (Scott and Novais, 2016). The first-line treatment for LCL is most commonly intralesional antimonials and amphotericin B (McGwire and Satoskar, 2014). However, intravenous (IV) and intramuscular (IM) treatments are often recommended due to having cure rates greater than 90% (Alfahaad, 2020). Other non-pharmacological treatments include cryotherapy or heat therapy (McGwire and Satoskar, 2014). Despite self-healing and clinical cure, parasites and parasitic DNA can persist in the scars of healed lesions for many years (Schubach et al., 1998). This phenomenon can lead to resistance against re-infection or to the development of mucosal lesions and severe disease (Schubach et al., 1998).

### Immunology

**Figure 2:**
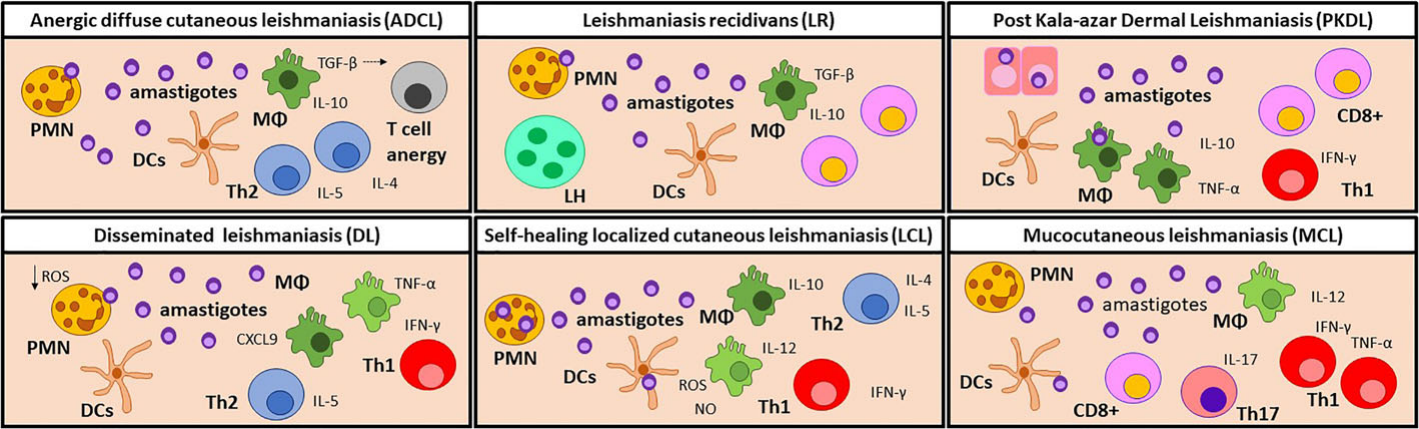
Immune cell profiles in the skin lesion of CL and PKDL patients. Anergic diffuse cutaneous leishmaniasis (ADCL) lesions are developed due to a lack of a Th1 response. Parasites can interact directly with macrophages which, even when abundant, are polarized towards an M2 profile and produce high levels of TGF-β. Consequently, T cells are activated towards IL-4, IL-5, and IL-10-producer Th2 cells. In this environment, T cells become anergic, leading to chronic disease. It is noteworthy that this manifestation is particularly common in immunosuppressed patients. Disseminated leishmaniasis (DL) patients show a temporary impairment of the early T cell response, which allows for dissemination of the parasite. Later, CXCL9 attracts T-cells to the lesion site, causing a lack of systemic Th1 response. In the lesion, IL-10, iNOS, TNF-α, and IFN-γ are found in similar levels as LCL lesions. In self-healing localized cutaneous leishmaniasis (LCL), phagocytes are the first responders against infection by producing ROS and NO to destroy the parasites, but can also become permissive hosts for amastigotes. A Th1 response, characterized by IL-12 and IFN-γ, is necessary for the control of the disease while the Th2 immune response promotes tissue repair, leading to the self-healing of the lesion. In rare occasions, LCL can relapse as Leishmaniasis recidivans (LR), but the trigger stimulus is still unknown. LR is characterized by TGF-β and IL-10 production, as well as an increased ratio of CD8+ vs. CD4+ T cells. LR lesions also show a granulomatous infiltrate containing lymphohistiocytic and multinucleated giant cells. Post Kala-azar Dermal Leishmaniasis (PKDL) develops when the cellular response is reactivated after drug treatment. The remaining parasites in the skin elicit an increased inflammatory response and the development of dermal lesions. An increased proportion of CD8+ T cells over CD4+T cells is often observed, but the role of CD8 T cells is still unclear. Mucocutaneous Leishmaniasis (MCL) lesions are characterized by a chronic and hyperactive inflammatory immune response. IFN-γ and TNF-α producing Th1 cells and cytotoxic CD8+ cells are predominant leading to tissue destruction. The high numbers of Th17 and polymorphonuclear neutrophils (PMN) contribute to exacerbated inflammation, while the low levels of IL-10 detected are related to the poor control of inflammation. Manifestations with a mixed immune profile lead to a moderate pathology, while a hyperpolarized response results in a severe disease. MΦ, Macrophages; DCs, dendritic cells; PMN, Neutrophils; Th, T helper cells; LH, lymphohistiocytic and multinucleated giant cells; PKDL, Post Kala-azar Dermal Leishmaniasis.

Whether or not LCL lesions self-heal or become chronic depends primarily on the initial immune response (Scott and Novais, 2016). The main characteristics of an immune response to LCL are summarized in **Figure 2**. Immediately after infection, *Leishmania* parasites are phagocytosed by immune cells recruited to the site of the infection, such as neutrophils, dendritic cells (DCs), and monocytes (Ribeiro-Gomes et al., 2012; Scott and Novais, 2016; Rossi and Fasel, 2018). Neutrophils play divergent roles during infection, as they can be leishmanicidal, but also function as Trojan horses for the parasites (Scott and Novais, 2016). For instance, neutrophil extracellular traps (NET)s have been shown to kill *L. amazonensis* promastigotes (Guimarães-Costa et al., 2009). On the other hand, phagocytosis of *L. major-*infected apoptotic neutrophils hinders the activation of macrophages and DCs, resulting in the persistence of the parasites (van Zandbergen et al., 2004). Although neutrophils have been canonically described as the first cells to be recruited after *Leishmania* infection, new evidence suggests that a population of Ly6C+ inflammatory monocytes migrate to inflamed tissues first. Some studies indicate that these monocytes can control *L. major* parasites through a quick release of reactive oxygen species (ROS) during phagocytosis, known as a respiratory burst (Goncalves et al., 2011; Ponath and Kaina, 2017). However, others have shown that Ly6C monocytes contribute to the pathogenesis of infection by serving as a reservoir for parasite proliferation and cell-to-cell transmission (Romano et al., 2017; Heyde et al., 2018). In the later stages of the infection, macrophages become the canonical host for *Leishmania* parasites (Scott and Novais, 2016).

While innate immunity plays an important role in the initial response against LCL, activation of the adaptive cell-mediated immune response is critical for disease resolution and development of long term immunity. Although a mixed T helper (Th) 1 and Th2 response has been observed during active infection, a robust Th1 immune profile is mainly responsible for clinical cure (Schriefer et al., 2008; Castellano et al., 2009). Interleukin (IL)-12 is pivotal to controlling *Leishmania* infection, as it promotes the Th1 response and induces the production of Interferon gamma (IFN-γ) (Okwor and Uzonna, 2016b). Peripheral blood mononuclear cells (PBMC)s from patients with an acquired immunity to LCL stimulated with *L. major* antigen showed an increase in IFN-γ and tumor necrosis factor (TNF)-α (Kemp et al., 1999). Both IFN-γ and TNF-α activate macrophages and promote ROS production to mediate *Leishmania* clearance (Rossi and Fasel, 2018). LCL caused by the Sri Lanka *L. donovani* strain is also characterized by significantly higher levels of IFN-γ and TNF-α in the cutaneous lesions, which are involved in both parasitic killing and tissue damage (Manamperi et al., 2017; Kariyawasam et al., 2018). This strong Th1 response led to resolution of the infection in Sri Lankan LCL, while an increase in the Th2 cytokine IL-4 was observed in lesions with a poor response to treatment (Manamperi et al., 2017).

Nitric oxide (NO) and ROS act as the two main microbicidal mediators involved in controlling *Leishmania* infection. Phagocytic cells can mediate anti-parasitic activity quickly during the initial phases of infection, even in the absence of activation, by producing ROS (Novais et al., 2014). These molecules play an especially important role in *L. braziliensis* infection, as *L. braziliensis* amastigotes are known to be susceptible to ROS and they survive and replicate particularly well in the absence of ROS (Novais et al., 2014). Inducible NO synthase (iNOS) induces production of NO in macrophages following activation by IFN-γ and TNF-α, and ultimately mediates killing of *Leishmania* parasites (Castellano et al., 2009; Scott and Novais, 2016). Studies have demonstrated that mice lacking iNOS fail to control *L. major* infection, despite mounting a greater Th1 response than wild-type mice. NO, along with O2-, are the predominant molecules released by macrophages in the respiratory burst to kill *Leishmania* parasites in both mice and humans (Wei et al., 1995; Carneiro et al., 2016).

The induction of both a Th1 and Th2 response in LCL is supported by a study showing high levels of IFN-γ, TNF-α, IL-12, IL-10, and IL-4 in PBMCs culture supernatants from patients with active lesions (Castellano et al., 2009). An early Th2 response allows for the persistence of the parasitic infection through the production of its characteristic cytokines: IL-4, IL-10, and IL-13 (Rossi and Fasel, 2018). Studies show a decrease in both IL-10 and IL-4 after clinical cure of LCL lesions, suggesting these cytokines are involved in exacerbation of the disease and promotion of parasite proliferation (Schriefer et al., 2008; Castellano et al., 2009). IL-4 inhibits the production of IFN-γ and the differentiation of Th1 cells, while IL-10 inhibits IFN-γ-induced activation and leishmanicidal activity of macrophages (Schriefer et al., 2008; Castellano et al., 2009). Low levels of IFN-γ and high levels of IL-10 in the blood have been observed during the early stages of *L. braziliensis* infection. However, as the infection progresses, IL-10 levels decrease and IFN-γ concentrations increase in every clinical form caused by this *Leishmania* species (Schriefer et al., 2008; Oliveira et al., 2014). These findings indicate that the balance between anti-inflammatory and pro-inflammatory cytokines is the key to the full resolution of LCL lesions. For instance, studies demonstrate that as the levels of IL-10 increased in the PBMC of patients with LCL, TNF-α also increases, maintaining a balance between the two responses, which is not only necessary for controlling infection but also for preventing immunopatholgy (Gaze et al., 2006).

While a Th1 response is crucial to control the infection, Th2 cytokines promote tissue regeneration and reduce the severity of inflammation during lesion healing (Maspi et al., 2016). Increased levels of IL-10 can suppress the effects of IFN-γ, as high levels of IFN-γ can lead to tissue damage (Rocha et al., 1999). Furthermore, IL-4 and IL-13 can induce arginase-1 (Arg-1), a Th2 enzyme involved in tissue repair (El Kasmi et al., 2008). The activity of Arg-1 results in the generation of ornithine, which is converted to polyamine and proline resulting in collagen formation and tissue regeneration (Morris, 2007). Taken together, these observations suggest that both Th1 and Th2 responses are crucial to the self-healing of LCL lesions, as a Th1 immune response is critical for eradication of the parasites, while a Th2 immune response aids in tissue repair and preventing immunopathology.

Patients with LCL also show increased production of IL-17 by PBMCs, compared to healthy controls (Oliveira et al., 2014). IL-17 is a pro-inflammatory cytokine that promotes neutrophil recruitment and is involved in immunity to intracellular pathogens. Expression of IL-17 is observed in the lesions of LCL patients, suggesting its role in immunity, but also in promoting tissue damage and lesion formation. In *L. braziliensis* infection, IL-17 and TNF-α production is reduced by IL-10 and transforming growth factor (TGF)-β, which can inhibit inflammation and tissue destruction (Oliveira et al., 2014).

The persistence of a small number of *Leishmania* parasites in the dermis after healing of the lesion is due to the IL-10 mediated suppression of acquired immunity by a population of IL-10/IFN-γ dual producers CD4+CD25- Foxp3- Th1 cells (Trinchieri, 2001; Belkaid et al., 2002; Anderson et al., 2007). The continued presence of these parasites maintains a population of *Leishmania*-specific memory T cells (Peters et al., 2014). Interestingly, the Th1 response to *Leishmania* can also result in the production of effector memory T cells that provide immunity even in the absence of any remaining parasites (Colpitts et al., 2009). Previous research suggests that CD8+ T cells are important for the immune response to re-infection with *Leishmania*, however, others have reported that CD8+ T cells have a limited role in the secondary immune response (Okwor et al., 2014; Scott and Novais, 2016). Secondary immunity was found to be dominated by CD4+ T cells in LCL (Okwor et al., 2014).

It is important to mention that although self-healing is common in LCL, evasion of the immune system by parasites can lead to more severe manifestations of CL, as described below.

## Leishmaniasis Recidivans (LR)

### Epidemiology

Leishmaniasis recidivans (LR) is a rare chronic form of CL occurring in only about 3-10% of patients. It is characterized by the formation of relapsing papules around or within a scar from a previously healed acute lesion (Faccini-Martínez and Falqueto, 2016). These papules or nodules generally appear within months to years after healing of the primary lesion and do not progress to ulcers (Bittencourt et al., 1993; Goto and Lindoso, 2010). However, recurrence of LR following LCL has occurred even after 43 years following initial LCL infection (Marovich et al., 2001). In the Old World, namely Ethiopia, India, and Pakistan, LR is primarily caused by *L. tropica*, and rarely by *L. major* (Marovich et al., 2001; Stefanidou et al., 2008; Sharifi et al., 2010). In the New World, namely Brazil, however, LR cases are less frequent and have been due *to L. braziliensis, L. amazonensis, L. panamensis*, and *L. guyanensis* (Calvopina et al., 2006; Gangneux et al., 2007) (**Table 1**). Unlike acute lesions, diagnosis of LR can be difficult due to absence of parasites in lesion biopsies. Therefore, PCR must be used to identify the causative *Leishmania* species (Oliveira-Neto et al., 1998).

### Immunology

The main characteristics of an immune response to LR are summarized in **Figure 2**. Patients with LR display a strong hypersensitivity response to *Leishmania* antigen (Calvopina et al., 2006). Furthermore, LR lesions possess a granulomatous infiltrate containing lymphohistiocytic and multinucleated giant cells (Calvopina et al., 2006; Stefanidou et al., 2008; Ekiz et al., 2015). Since new LR lesions occur proximally to the location of the cured acute lesion, it is likely that the parasites persist at the site of initial infection and are reactivated by an unknown stimulus. One study found that, during *L. braziliensis* infection, *Leishmania* DNA located in the nasal mucosa resulted in an increased occurrence of LR (36.4% of positive patients) (Canário et al., 2019). It has also been hypothesized that local trauma or treatment with corticosteroids can contribute to LR relapse, possibly due to the induction of the wound-healing immune response, which provides a favorable anti-inflammatory environment for the parasite (Wortmann et al., 2000; Gangneux et al., 2007).

Since resistance to *Leishmania* is linked to a strong Th1 inflammatory immune response and subsequent macrophage activation to clear the intracellular parasites, it has been proposed that LR occurs due to putative defects in the cellular immunity of the host (Dassoni et al., 2017). For instance, anti-inflammatory cytokines IL-10 and TGFβ have been shown to mediate persistence of chronic infection in C57BL/6 mice after resolution of initial infection with *L. tropica*, the species most responsible for recurring LR (Anderson et al., 2008). However, this correlation has not yet been documented in human LR. Nonetheless, these experimental findings suggest that *Leishmania* parasites are never completely eliminated from the host, and they continue to persist and maintain immunity. One study reported a decreased ratio of CD4+ to CD8+ T lymphocytes in the peripheral blood during LR (Jaroskova et al., 1986). Conversely, resolution of LR after combination therapy is associated with an increase in CD4+ T lymphocyte proliferation, as well as IFN-γ production. Interestingly, this trend was enhanced when CD8+ cells were depleted in PBMCs, suggesting that CD8+ cells may also play a suppressive role in LR (Marovich et al., 2001).

## Mucocutaneous Leishmaniasis

### Epidemiology

Mucocutaneous leishmaniasis (MCL) is a rare clinical manifestation caused by the progression of CL into a more severe disease (David and Craft, 2009). In the New World, *Leishmania* parasites of the *Viannia* subgenus, such as *L. braziliensis*, *L. panamanensis*, *L. guyanensis*, and *L. amazoniensis*, can metastasize and cause MCL lesions (Sabzevari et al., 2020). Of these species, *L. braziliensis* accounts for most of the cases of MCL in South America (Amato et al., 2008). MCL endemic areas in the New World include Bolivia, Peru and Brazil (World Health Organization 2021c). MCL is less common in the Old World, but has been reported in Iran and certain areas of East Africa, especially Ethiopia and Sudan, and is caused by *L. major*, *L. tropica*, *L. aethiopica*, *L.donovani* and *L. infantum* (Strazzulla et al., 2013; Sunyoto et al., 2018; Sabzevari et al., 2020) (**Table 1**).

MCL metastatic lesions can appear simultaneously with CL lesions, or develop months to years after the resolution of primary lesions, ultimately leading to a progressive destruction of the mucosal tissue of the nose, mouth, and throat (Handler et al., 2015). Tissue necrosis results in facial disfiguration, which attracts stigma and marginalization in affected communities. Destruction of the mucosal membranes can also progress into life threatening disabilities that can prevent the patient from working and earning a living (World Health Organization 2021c).

Development of MCL occurs in up to 20% of all CL patients, and can depend on the size and number of cutaneous lesions, the parasite species and virulence, the host’s immune response, and the patient’s sex, age, and nutritional status (Machado-Coelho et al., 2005; David and Craft, 2009). Furthermore, there is evidence suggesting that RNA viruses that infect *Leishmania* can favor development of MCL and predict failure of treatment. These viruses have been abundantly found in human MCL lesions, but were infrequent in CL lesions (Zangger et al., 2013). In particular, *Leishmania* RNA Virus 1 (LRV1), harbored by *L. guyanensis* and *L. braziliensis*, has been shown to promote autophagy in the host and leads to disease exacerbation and ultimately to the development of MCL (Olivier and Zamboni, 2020).

MCL lesions can develop upon the direct bite of a sand fly on the mouth or nose, or as a consequence of proximal facial skin lesions, as seen during *L. major* infection. They can also arise at later time points from different parts of the body, as with parasites of the *L. braziliensis* complex. In this instance, new or latent parasites are likely to spread from one location to another *via* the blood or lymphatic system (Strazzulla et al., 2013). The *Leishmania* species capable of causing MCL have to overcome a crucial challenge: adapting to survive and persist in the mucosal membranes. It has been hypothesized that some species, such as *L. infantum*, can acquire the temperature resistance needed to survive in the mucosa, and travel through the blood stream to reach a distal mucosal membrane and establish a new infection (Aliaga et al., 2003; Faucher et al., 2011; Cocuzza et al., 2013).

### Immunology

The key feature of MCL, which distinguishes it from other forms of leishmaniasis, is chronic and elevated inflammation and hypersensitivity (**Figure 2**). In contrast to LCL, a Th1 and CD8+ T cell-polarized response in MCL can become detrimental, as it can lead to cytotoxicity and tissue destruction. While severe disease in many forms of CL is driven by high parasitic burdens and a limited Th1 response, exacerbation of MCL is mediated by immunopathology and an exaggerated cellular response (Scott and Novais, 2016).

PBMCs from MCL patients show elevated IFN-γ and TNF-α production and lower IL-10 levels after antigen stimulation, compared to patients without mucosal involvement (Bacellar et al., 2002; Nylén and Eidsmo, 2012). This strong Th1-polarization is characterized by a delayed-type hypersensitivity response, and low numbers of parasites in the lesions (Scott and Novais, 2016). Another study demonstrated an increase in lymphocyte activation in MCL patients, presented by higher frequencies of CD4+ CD62L–, CD4+ CD69+, CD8+ CD69+, and CD4+ CD28– T cells compared to patients with other forms of CL (Gaze et al., 2006). In particular, CD4+ CD28– T cells form a population of chronically activated lymphocytes, implicated in promiscuous cytotoxicity and co-stimulation (Gaze et al., 2006). Furthermore, MCL patients display a higher percentage of IFN-γ and TNF-α-expressing CD4+ T cells in their blood, and lower levels of IL-10-producing regulatory monocytes compared to patients without mucosal involvement (Gaze et al., 2006). MCL lesions also show high levels of Th17 cells and neutrophils, as well as increased expression of IL-17 (Boaventura et al., 2010; Borges et al., 2018).

PBMCs from MCL patients exhibit increased cytolytic properties compared to those from patients with other forms of CL and healthy controls (Brodskyn et al., 1997). For instance, as disease severity progresses in *L. braziliensis* patients, the percentage of CD8+ over CD4+ T cells increases. These CD8+ have cytotoxic activity characterized by granzyme A expression (Faria et al., 2009). Cytotoxicity is the key feature in *L. braziliensis* lesions, as highlighted by a transcriptional profiling study, and it occurs concurrently with skin destruction in humans (Novais et al., 2015). Along with CD4+ and CD8+ cells, MCL lesions display increased number of CD68+ macrophages, and elevated levels of IFN-γ (Dutra et al., 2011). Another possible mechanism for apoptosis in the skin is mediated by Foxp3+ T reg cell, positively correlated with an upregulation of FasL and active caspase-3 in MCL lesions (Carneiro et al., 2009). Finally, antibody responses are also higher in MCL, compared to patients without mucosal involvement, due to an accumulation of B and plasma cells in the lesions. These cells have also been implicated in contributing to tissue damage (Nylén and Eidsmo, 2012).

Interestingly, *Leishmania* virus LRV1 can also induce pro-inflammatory cytokines and chemokines that contribute to tissue destruction and disease exacerbation (Rossi and Fasel, 2018). LRV1 activates the TLR3/TRIF signaling pathway in macrophages, leading to the production of Type I IFNs and mediating autophagy, a process known to inhibit inflammosome activation, important for parasitic clearance. MCL lesions show lower levels of inflammosome products, such as IL-1β and cleaved caspase-1, compared to lesions of patients with other forms of CL (Olivier and Zamboni, 2020).

A balance between Th1 and Th2 responses is crucial to maintain homeostasis and promote wound healing and tissue regeneration. With a hyperactive Th1 response, PBMCs from MCL patients display a limited response to IL-10 stimulation, possibly mediated by lower expression of the IL-10 receptor in the lesions, compared to LCL patients (Bacellar et al., 2002; Faria et al., 2005).

## Anergic Diffuse Cutaneous Leishmaniasis (ADCL)

### Epidemiology

Another less common manifestation of CL is Anergic Diffuse Cutaneous Leishmania (ADCL), which is caused by *L. aethiopica* in the highlands of Ethiopia and Kenya, but also reported in Namibia and Tanzania (Walton and Velasco, 1989); and by *L. amazonesis* and *L. mexicana* throughout the Americas in countries such as Ecuador, Venezuela, Brazil, Dominican republic, Mexico, Honduras, Nicaragua, Peru, Bolivia, Colombia and the United States of America (southern Texas) (Walton and Velasco, 1989; Pearson and Sousa, 1996; Silveira et al., 2004; Reithinger et al., 2007; Hashiguchi et al., 2016) (**Table 1**). ADCL is particularly prevalent in immunocompromised patients, such as those infected with HIV (Amato et al., 2004).

ADCL starts as a papule, usually on the extremities or the face, and without the development of ulcers. The spread of ADCL is slow but can persist for decades, leading to the development of further papules and tubercules (Pearson and Sousa, 1996). Unlike other forms of *Leishmania* infection, parasites disseminate through the peripheral lymphohaematogenic route in ADCL (Silveira, 2019), promoting a chronic disease refractory to standard treatments (Amato et al., 2004). Interestingly, the Delayed-type Hypersensitive (DTH) skin test is negative in the majority of ADCL patients, indicating a suppression of the Th1 response (Silveira et al., 2004).

### Immunology

The main features of an immune response to ADCL are summarized in **Figure 2**. The nodules contain a large number of macrophages infected with *Leishmania* amastigotes, but there is a noticeable lack of T cells (Silveira et al., 2004). Immunologically, ADCL is characterized by T cell anergy, which is the key feature that distinguishes ADCL from other forms of CL (Reithinger et al., 2007). One proposed mechanism behind T cell anergy is an excessive release of TGF-β by macrophages. TGF-β is an immunosuppressive and pro-apoptotic cytokine, and high TGF-β levels suppress CD4+ T cells responses during *L. amazonesis* infection (Pinheiro et al., 2004). Macrophages are not the only source of TGF-β and other cells may be concurrently involved in the apoptosis observed in ADCL (Pinheiro et al., 2004).

Individuals with ADCL also show high levels of Th2 cytokines, such as IL-4, IL-5, and IL-10, but low levels of Th1 cytokines, such as IFN-γ, in lesion biopsies (Silveira et al., 2004). These observations indicated that despite T cell anergy, ADCL patients display enhancement of a Th2 response, which polarizes macrophages towards a *Leishmania-*permissive M2 anti-inflammatory phenotype (Pinheiro et al., 2004). Increased Th2 cytokine production is also associated with decreased IFN-γ levels, which consequently impairs macrophage activation and leads to clinical progression of ADCL (Silveira et al., 2004).

Surface antigens on *Leishmania* parasites also contribute to this unbalanced cell mediated response (Nogueira et al., 2016). A specific *Leishmania* antigen important for ADCL is LPG, which interacts with TLR4 on antigen presenting cells, altering their phenotype to promote differentiation of Th2 CD4+ T cells (Silveira, 2019). This is mediated by a reduction of NO and pro-inflammatory cytokines, such as IFN-γ (Nogueira et al., 2016). *Leishmania* species such as *L. amazonesis* also inhibit activation of macrophages by interacting with TLR9 (Silveira et al., 2004). TLR9 activation increases the expression of CD200, an immunosuppressive glycoprotein that downregulates NO production in the few macrophages still activated during the ADCL immune response. This likely contributes to the strong Th2 response observed in patients, ineffective against *Leishmania* parasites (Sauter et al., 2019).

## Disseminated Leishmaniasis (DL)

### Epidemiology

Disseminated leishmaniasis (DL) is characterized by the presence of multiple lesions in two or more non-contiguous body regions. These lesions may be a mixture of acneiform, papular, nodular, and ulcerative types with involvement of the nasal mucosa in up to 44% of DL patients (Turetz et al., 2002; Machado et al., 2011). An increase in epidermal hyperplasia and the presence of a follicular pattern in the acneiform type lesion has been observed, unlike classical LCL (Carvalho et al., 1994). Parasites from the original infection location spread quickly to distal sites and produce DL lesions within days to weeks of infection (Turetz et al., 2002). It should be noted that DL is distinct from ADCL, as DL patients exhibit a strongly positive Leishman skin test, present few parasites at the lesion site, and develop lesions that often ulcerate, unlike ADCL, as described above (Hashiguchi et al., 2016).

DL is mainly endemic in the New World, almost exclusively in northern and northeastern Brazil, and is caused primarily by *L. braziliensis* (Costa et al., 1986; Galvão et al., 1993; Membrive et al., 2017). However, *L. amazonensis* and *L. panamensis/guyanensis* have also been reported as the causative agents of DL in Brazil and Colombia (Carvalho et al., 1994; Hashiguchi et al., 2016; Cataño and Pinzón, 2019; Ovalle-Bracho et al., 2019) (**Table 1**). DL has been identified as an emerging disease in northeastern Brazil and constitutes 1.9% of all CL cases, compared to 0.2% in the past decades (Jones et al., 1987; Turetz et al., 2002). Increases in DL incidence precede a similar upsurge in American Tegumentary Leishmaniasis (ATL) cases by about 2 years. Thus, it is hypothesized that the factors affecting DL transmission respond more quickly to environmental changes than CL and ML (Schriefer et al., 2009). Those with the highest risk of developing DL, compared to LCL, are males above 19 years of age in agricultural occupations (Turetz et al., 2002).

### Immunology

The ability of the parasite to disseminate rapidly to other locations of the skin and mucosa, the absence of lymph node enlargement, and prodromal symptoms such as fever and malaise, suggest that *Leishmania* is disseminated through the bloodstream during DL (Turetz et al., 2002). Furthermore, it has been proposed that proliferation of blood vessels mediates inflammation during DL and enhances growth and survival of the parasite (Mendes et al., 2013). Both host and parasite factors are hypothesized to play roles in promoting parasite dissemination in DL. The main immunological features of DL are summarized in **Figure 2**.

Similar to LCL, lesions from patients with DL are characterized by mononuclear cell infiltration as well as granuloma formation (Carvalho et al., 1994). In particular, inflammatory infiltrates in the non-ulcerative DL lesion biopsies were primarily comprised of macrophages, plasmacytes, T cells, and B cells (Mendes et al., 2013). However, DL biopsies contained lower numbers of B cells compared to those of LCL (Vieira et al., 2002). This decreased number of B cells may explain the presence of non-ulcerative lesions in DL, since B cells have been linked to tissue damage during *Leishmania* infection (Nylén and Eidsmo, 2012). On the other hand, DL patients exhibit higher antibody titers to *Leishmania* antigen than LCL, with increased antibody levels correlating to higher mucosal involvement (Carvalho et al., 1994).

Neutrophils are another important cell type in this form of leishmaniasis, since they are the first responders to infection with *Leishmania* and have been linked to pathogenesis of infection (Peters et al., 2008). It has been shown that *L. braziliensis* isolates from DL patients infect neutrophils at a lower frequency than *L. braziliensis* isolates from LCL patients. Furthermore, these DL-infected neutrophils contain lower intracellular amastigote numbers than those of LCL. However, neutrophils from *L. braziliensis* DL isolates also display decreased activation markers and oxidative burst compared to neutrophils infected with isolates from LCL patients. This data suggests that increased production of reactive oxidants by neutrophils is not responsible for control of the parasite in DL (Conceição et al., 2016; Cardoso et al., 2019).

Previous data suggests that parasite dissemination in DL is due to a lack of peripheral Th1 immune response. Following *Leishmania* antigen stimulation, PBMCs from *L. braziliensis* DL patients produce less IFN-γ and TNF-α but similar levels of IL-5 and IL-10 compared to PBMCs from CL patients. This observation is intriguing since antigen produced from *L. braziliensis* parasites isolated from DL patients stimulated higher production of IFN-γ and TNF-α in PBMCs from both CL and DL patients, than *L. braziliensis* antigen from LCL patients (Turetz et al., 2002; Leopoldo et al., 2006). Although DL patients produce less TNF-α than LCL and MCL patients, DL patients with mucosal lesions exhibited increased TNF-α production by PBMCs compared to those without mucosal involvement (Machado et al., 2011). Similar to PBMCs from DL patients (Leopoldo et al., 2006), biopsies from DL patients have also showed lower expression of IFN-γ compared to individuals with LCL. However, the frequency of TNF-α and iNOS producing cells as well as CD68+ macrophages remained the same between these DL and LCL biopsies (Vieira et al., 2002).

A study by Machado et al., investigating the *in situ* and systemic response to *L. braziliensis–*caused DL provided more clarity behind this immune response by evaluating cytokine and chemokine production. They found that peripheral production of IFN-γ and TNF-α was reduced in PBMCs as reported previously (Leopoldo et al., 2006; Machado et al., 2011). Yet, *in situ* production of IFN-γ, TNF-α, and IL-10 in papular and ulcerated lesions of DL and classical American cutaneous leishmaniasis (ACL) patients was comparable. Moreover, serum levels of CXCL9, a T cell chemoattractant, was higher in DL than ACL patients, suggesting that *Leishmania*-reactive T cells migrate to the lesion site (Machado et al., 2011). This migration is likely to reduce T cells in the peripheral blood, possibly explaining why PBMCs from *L. braziliensis* DL patients produce less IFN-γ and TNF-α, while maintaining a positive T cell response against *Leishmania* antigen in the tissue. In contrast to *L. braziliensis*-caused DL infection, one study found that DL patients infected with *L. amazonensis* exhibited decreased CD4+ cells and a lack of T cell response to *Leishmania* antigen, yet these abnormalities were restored after successful treatment (Carvalho et al., 1994).

Collectively, these observations suggest that a temporary impairment of the early T cell response, as well as peripheral Th1 response, allows for dissemination of the parasite, but a later immune response in the tissue is able to control the infection. This may be the reason for the low parasite numbers observed in DL lesions, as well as presentation of ulcers similar LCL due to a delayed inflammatory response at the lesion site (Machado et al., 2011). Although pro-inflammatory cytokines allow for defense against the parasite, they also contribute to the formation of ulcers (Machado et al., 2002). However, the development of DL is not linked to immune suppression of the host, since it has been also observed in young and immunocompetent individuals (Jirmanus et al., 2012).

Although the exact mechanism by which *L. braziliensis* generates different clinical forms is unknown, genetic variability between *L. braziliensis* strains is thought to play a role in the different disease outcomes in *L. braziliensis* infection. Randomly amplified polymorphic genetic markers was used to distinguish parasite isolates and define five separate clades of *L. braziliensis* in Corte de Pedra. DL patient isolates were enriched in two of these distinct clades based on RAPD profile, designated clades A and D, in comparison to LCL and mucosal isolates (Schriefer et al., 2004; Schriefer et al., 2009). A further study revealed an association between single-nucleotide polymorphisms, insertions-deletions, and certain haplotypes of *L. braziliensis* with increased risk of developing DL (Queiroz et al., 2012). Moreover, geographic distribution of DL in this area correlated with the *L. braziliensis* genotype clades associated with DL. This observation further supports the notion that certain parasite strains account for active DL foci (Schriefer et al., 2009).

## Post Kala-Azar Dermal Leishmaniasis

### Epidemiology

Post Kala-azar dermal leishmaniasis (PKDL) is a dermatological complication of VL observed in some patients years after completion of WHO recommended therapy, especially in two specific regions (**Table 1**) with different manifestations of the disease (**Table 2**) (McGwire and Satoskar, 2014). In South Asia, PKDL manifests as papulonodular lesions (polymorphic), while in East Africa it causes hypomelanotic (macular) lesions (Ramesh et al., 2015; Chatterjee et al., 2020). Although PKDL is not life threating, it negatively affects the quality of life of the patients (Pal et al., 2017), and it represents a socioeconomic burden in the endemic regions (Mukhopadhyay et al., 2014).

**Table 2:** Comparison between PKDL in the two main endemic regions in the Old World.

Geographic region	East Africa	South Asia
**Countries affected**	Mostly Sudan, but also Ethiopia, Kenya and Uganda	India, Nepal and Bangladesh
**Most common dermal manifestations**	Macular	Polymorphic and macular
**Incidence within VL patients**	50-60%	5-10%
**Time of appearance after VL**	1 year after treatment or concomitant	2-3 years after treatment
**Self-healing lesions**	Yes (within 1 year)	No
**Parasitic burden in lesions**	Lower	Higher
**Cell infiltrate**	Scarce and patchy	Dense and diffuse

PKDL, Post Kala-azar Dermal Leishmaniasis; VL, Visceral Leishmaniasis.

In the South Asian region (India, Nepal, Bangladesh), 5-10% of the patients with supposedly cured VL develop PKDL 2-3 years after treatment (Burza et al., 2018; Rijal et al., 2019). Whereas, in East Africa (primarily in Sudan, but also Ethiopia, Kenya and Uganda) the incidence is 50-60% and the clinical manifestations appear either after VL remission or even during the treatment (Zijlstra and el-Hassan, 2001; Zijlstra and Alvar, 2012; Zijlstra et al., 2020). In both regions, the relapse time depends on the treatment regimen (Zijlstra and el-Hassan, 2001; Rijal et al., 2019; Goyal et al., 2020; Zijlstra et al., 2020). The immune responses to PKDL in Asia and Africa are different. Approximately 85% of cases in Africa show self-healing lesions, but lesions rarely resolve in the Asian PKDL (Burza et al., 2018). However, it is noteworthy that *L. donovani* parasites are genetically varied depending on the geographical regions (Zemanová et al., 2004; Lukes et al., 2007).

Environmental and host related factors can favor the transition to PKDL, such as the exposure to UV light, genetic polymorphisms and even genetic changes of the parasite itself. It is been hypothesized that UV light might contribute to the development of PKDL (Mukhopadhyay et al., 2014). UV can induce inflammation and damage in skin cells affecting the function of antigen presenting cells, particularly Langerhans cells, leading to the generation of suppressor T cells and altered cytokine production, hence disrupting cellular immunity resulting in replication of parasites in the dermis (Clydesdale et al., 2001; Seité et al., 2003). Nevertheless, this hypothesis implies that the sun-exposed areas of the body would be the ones affected by UV light, which does not match the all-over the body distribution observed in macular cases by Sengupta et al., 2019b.

A polymorphism in the IFN-γ receptor gene (*IFNGR1*) is associated with an increased risk of developing PKDL. Interestingly, it does not increase susceptibility to VL, which is linked to a polymorphism in *IL-4* gene (Mohamed et al., 2003; Salih et al., 2007). Genetic changes in *L. donovani* have also been identified and likely contribute to development of PKDL. Parasites isolated from VL and PKDL patients are consistently identical among groups, but it is possible that parasites have gained genetic modifications defined by the stage of the disease (PKDL vs. VL) and geographical region (Sreenivas et al., 2004; Dey and Singh, 2007). Genetic factors from the host and the parasite might play a role in susceptibility and development of PKDL after VL.

Even though PKDL is not lethal, it increases the spread of VL (Le Rutte et al., 2019). Patients’ skin lesions serve as reservoirs for *L. donovani* by making the parasites accessible to the sandflies during blood meals (Molina et al., 2017; Mondal et al., 2019). In fact, Chapman et al estimated that PKDL could have contributed up to 25% of VL cases in a Bangladesh population (Chapman et al., 2020).

### General Immune Response

In general, the development time of PKDL after VL remission induces different immune profiles. The main characteristics of this immune response are summarized in **Figure 2**. Asian PKDL is more likely to become chronic and is associated with CD8+ T cell infiltration into the cutaneous lesions, while Sudanese PKDL appears sooner after remission of VL and is characterized by the reactivation of the immune response (Mukhopadhyay et al., 2014).

In PKDL, the Th1 response increases as a direct result of treatment, but it can also occur spontaneously in some instances, and is believed to contribute to disease pathogenesis (Zijlstra and Alvar, 2012). The successful treatment of VL reactivates the cellular immune response (Gasim et al., 2000), characterized by a decrease in regulatory T cells, TGF-β and IL-10 levels, and an increase in IFN-γ, TNF-α, and IL-12 (Mondal et al., 2010; Katara et al., 2011). *L. donovani* persisting in the skin is detected by re-activated immune cells, which infiltrate into the cutaneous tissue causing dermal inflammation. *Leishmania*-reactive T cells from peripheral blood also migrate to the skin and produce IFN-γ, exacerbating the inflammatory response and giving rise to the dermal manifestations characteristic of PKDL (Ismail et al., 1999; Gasim et al., 2000).

### Immunology of South Asian PKDL

Patients can be categorized into two different subgroups: polymorphic and monomorphic PKDL, depending on the characteristics of the lesions. In polymorphic PKDL is characterized by the development of papules and/or nodules, as well as hypopigmented macules. In monomorphic PKDL, patients develop only one type of lesion, mostly papulonodes (Zijlstra and Alvar, 2012; Kaushal et al., 2016). Even though polymorphic PKDL has been previously recognized as the most common manifestation (Zijlstra and Alvar, 2012), a recent surveillance study in Bengal indicates that the proportion might be equal for both manifestations (Sengupta et al., 2019a).

Histologically, the polymorphic lesions are characterized by the presence of dense and diffuse dermis cell infiltrate comprised of elevated numbers of macrophages and B cells, but fewer DCs and Langerhans cells (Mukherjee et al., 2015; Mukherjee et al., 2019; Sengupta et al., 2019b). The increase in macrophages is associated with an enhanced expression of IL-10, IL-12p40 (Mukherjee et al., 2015), M2-macrophage markers, and a reduction of TLR2/4 expression as well as ROS, and NO production (Mukhopadhyay et al., 2015), which constitutes a favorable milieu for parasite replication. In the lesions of monomorphic PKDL, the cell infiltrate is patchy and less abundant. In contrast with the polymorphic manifestation, monomorphic PKDL is characterized by a reduction of *Leishmania*-Donovan bodies (reflecting a lower parasitic load), DCs, CD4+ T-cells, and macrophage infiltrates (Sengupta et al., 2019b).

Polymorphic PKDL lesions show a higher parasitic burden, as well as higher levels of IFN-γ and TNF-α, compared to monomorphic lesions (Kaushal et al., 2016; Sengupta et al., 2019b). In terms of cellular infiltration in the lesion, polymorphic patients show an increase of CD3+ T cells and NK cells compared to the naïve group, whereas the monomorphic group is comparable to the naïve. The higher lymphoproliferation in the polymorphic PKDL patients can be explained by the higher number of circulating parasites, which also correlates with increased IFN-γ and TNF-α levels (Kaushal et al., 2016). A higher proportion of CD8+ cells over CD4+ T cells in dermal lesions is often reported in both variants, but is more elevated in the polymorphic one (Mukherjee et al., 2019; Sengupta et al., 2019b). In particular, high numbers of CD8+ CCR4+ cells are attracted to the dermal lesion site by the chemokines CCL17 and CCL22 expressed in the lesions (Mukherjee et al., 2019). Although the role of CD8+ cells in PKDL pathogenesis has yet to be clarified, one study reported increased levels of granzyme B, associated with cytotoxic lymphocyte responses, and a higher percentage of CD8+ CD64+ and CD4+ CD64+ T cells in the blood compared to naïve patients (Kaushal et al., 2016). In contrast, another study showed a reduction in the cytotoxic response, characterized by a decrease in Granzyme B, perforin, Zap70, and PD1 expression during PKDL, which reflects the exhaustion of CD8+ cytotoxic cells associated with disease progression. It is noteworthy that the suppressive profile was reverted after treatment with sodium antimony gluconate and miltefosine (Mukherjee et al., 2019).

The Th17 response is also increased during PKDL. In particular, *Il-17*, *Il-23* and *RORγt* mRNA levels were increased in the lesions during infection, and were restored to control levels after treatment (Katara et al., 2012). High levels of IL-17 lead to increased production of NO and TNF-α in PBMCs from the same patients, promoting a sustained inflammatory response (Katara et al., 2012).

Circulating levels of CXCL13, a B-cell recruiting chemokine, were also elevated in PKDL lesions, compared to healthy samples. This resulted in B cell numbers being increased 6.5-fold in polymorphic and 4-fold in monomorphic PKDL lesions (Sengupta et al., 2019b). Antibody production by B cells has been hypothesized to promote an immunosuppressive environment in PKDL (Sengupta et al., 2019b), as *Leishmania* parasites can directly ligate to IgG FC gamma receptors on infected macrophages to induce the production of IL-10 (Sutterwala et al., 1998; Kane and Mosser, 2001). Nevertheless, no conclusive data has been published to this date regarding this particular role for B-cells.

### Immunology of African PKDL

In East Africa, specifically Sudan, but also Ethiopia, Kenya and Uganda, the most common manifestation of PKDL is the macular form (Zijlstra and Alvar, 2012). Less information is available regarding Sudanese PKDL compared to the Asian disease, nevertheless, some differences have been documented. The Sudanese population displayed a high *Leishmania-*specific cellular response, which is observable only until the patients start developing PKDL. This response is characterized by an increase of PBMC proliferation and IFN-γ production (Gasim et al., 2000). Development of PKDL depends on the reactivation of the immune system and IFN-γ production against skin parasites. IFN-γ appears to be the main cytokine involved in PKDL development, as levels of IL-4, IL-5 and IL-10 were not different between patients who developed the cutaneous manifestation after VL and those who did not (Gasim et al., 2000).

## Conclusion and Final Remarks

Infection with *Leishmania* results in a broad spectrum of clinical manifestations, from the development of self-healing cutaneous lesions to more destructive infections. Together with the causative *Leishmania* strain, the immune response is the cornerstone that defines the pathology of the disease. Clearly, an exaggerated immune polarization, either towards a Th1 or Th2, leads to the development of a more severe disease, which is the case for MCL and ADCL, respectively. On the other hand, the balance between inflammatory and anti-inflammatory responses results in a more moderate disease, as in DL, and especially in LCL, where lesions are commonly self-healing (**Figure 1**).

First line treatments, such as pentavalent antimonials, amphotericin B, paromomycin, and miltefosine have demonstrated their effectiveness in parasite killing. However, numerous side effects such as cardiotoxicity, pancreatitis, nephrotoxicity, renal failure, teratogenicity, and impaired liver function in addition to the rise in parasitic resistance to these chemotherapies are associated with current therapies (Amato et al., 2007; Amato et al., 2008; Roatt et al., 2014). Hence, host directed therapies, which directly modulate the immune response against parasites could be novel treatments for different forms of CL. Efficacy of immunomodulators in the treatment of CL has not only been demonstrated in experimental animal studies (Buates and Matlashewski, 1999), but also in patients (Chouhan et al., 2014; Dos Santos et al., 2018; Caridha et al., 2019; Nahidi et al., 2021). Immunotherapy can also be a suitable option for patients with clinical resistance to recommended drugs; for example, patients from Peru with resistance to antimony showed a faster resolution of CL lesions after the use of the TLR7 agonist imiquimod (Miranda-Verástegui et al., 2005). Also, in MCL patients infected with *L. braziliensis*, the administration of a TNF-α inhibitor together with pentavalent antimony reduced the healing time of antimony alone, as well as reducing exposure to chemotherapy (Lessa et al., 2001; Machado et al., 2007).

It is worth mentioning that the major risk factors in developing all types of leishmaniasis are related to social conditions. Poverty leads to malnutrition, which could compromise immunity and lead to the development of a full-blown disease. Climate change, occupational exposure, and poor sanitary conditions expose people to a closer contact with the parasites in forested areas and migration of non-immune people to endemic areas favors the transmission cycles (Centers for Disease Control and Prevention, 2020).

Taken all together, these observations show that expanding the knowledge about the interplay between the parasite and the immune system is crucial for developing a comprehensive strategy to combat the disease, as it has provided a broad range of potential therapies to improve the treatment of leishmaniasis.

## Author Contributions

GV, TP-F, EH, NC, and BC wrote the paper. AS conceptualize and wrote the paper. All authors contributed to the article and approved the submitted version.

## Funding

Research in ARS lab is supported by funding from NIAID, Global Health Innovation Technology Fund and Wellcome Trust.

## Conflict of Interest

The authors declare that the research was conducted in the absence of any commercial or financial relationships that could be construed as a potential conflict of interest.
